# Proceedings of the 2011 MidSouth Computational Biology and Bioinformatics Society (MCBIOS) Conference

**DOI:** 10.1186/1471-2105-12-S10-S1

**Published:** 2011-10-18

**Authors:** Jonathan D Wren, Doris M Kupfer, Edward J Perkins, Susan Bridges, Stephen Winters-Hilt, Mikhail G Dozmorov, Ulisses Braga-Neto

**Affiliations:** 1 Arthritis and Immunology Research Program, Oklahoma Medical Research Foundation; 825 N.E. 13th Street, Oklahoma City, OK 73104-5005, USA; 2 Aerospace Medical Institute, Federal Aviation Administration, Oklahoma City, OK 73169, USA; 3 US Army Engineer Research and Development Center, 3909 Halls Ferry Road, Vicksburg, MS 39180, USA; 4 Department of Computer Science and Engineering, Mississippi State University, Box 9637, Mississippi State, MS 39762, USA; 5 Computer Science Department, University of New Orleans, USA; 6 Department of Electrical and Computer Engineering, Texas A&M University, College Station, TX 77843-3128, USA

## Introduction

The Eighth Annual Conference of the MidSouth Computational Biology and Bioinformatics Society (MCBIOS’2011) was held in College Station, Texas on April 1-2, 2011. The Conference General Chair was Ulisses Braga-Neto, the MCBIOS President for the 2010-2011 term, from Texas A&M University. There were nearly 200 registrants and 140 abstracts were submitted, divided into 48 oral presentation abstracts and 92 poster session abstracts.

In addition, participants attended talks by very distinguished Keynote speakers. Joan W. Bennett, from Rutgers University and Member of the National Academy of Science, presented the talk “Chromosomal Composition and Computational Competence;” Donald Geman, from The Johns Hopkins University and co-inventor of the Gibbs Sampler and Random Forest Classifiers, lectured on “Measuring Network Regulation and Differential Expression by Rank Conservation;” while John Weinstein, Chair of the Department of Bioinformatics and Computational Biology at the University of Texas M.D. Anderson Cancer Center, talked on “Personalizing Cancer Medicine in the Era of Next-Generation Sequencing: Omics and Informatics.”

The conference also benefited from invited dinner and lunch speakers, who gave informal, highly informative and entertaining talks. Edward R. Dougherty, from Texas A&M University and Director of Computational Biology at the Translational Genomics Institute, asked the audience “Is Biological Science Delightful?” whereas Ernesto Marques, from the Center for Vaccine Research at the University of Pittsburgh, gave the talk “Activation of the Complement System in Dengue Infection: Opportunities for Computational Modeling.”

Participants also had the opportunity to attend hands-on workshops on NCBI tools, presented by Peter Cooper from NCBI/NIH, and on protein evolution, presented by Hugh Nicholas and Troy Wymore, from the Pittsburgh Supercomputing Center. The winners of conference awards were:

Best Oral Presentations (students):

First place: Suzanne Matthews, Texas A&M University

Second place (3-way tie): Lori Dalton, Texas A&M University

Second place: Shelton Griffith, Oak Ridge National Lab

Second place: Winston Haynes, Hendrix College

Best Oral Presentations (Post-Doctoral fellows):

Yan Li, NCTR

Fan Zhang, IUPUI

Best Poster (Computation):

First place: Tianchuan Du, Southern University

Second place: Christopher Cathey, Jackson State University

Third place: Ralph Crosby, Texas A&M University

Best Poster (Biology):

First place: Awantika Singh, UALR/UAMS

Second place: Mohammed Shahrokh Esfahani, Texas A&M University

Third place: Fang-Han Hsu, Texas A&M University

**MCBIOS Outstanding Service Award**: Dr. Jonathan D. Wren, Oklahoma Medical Research Foundation

## Proceedings summary

Accepted for publication in the conference proceedings for this year were 21 research manuscripts out of a total of thirty three papers submitted for consideration (~64%). The volume of papers dropped a bit from last year’s record-setting forty-three papers submitted and twenty eight accepted [1-28], yet the acceptance rate was very similar. At least 2 reviewers, a mixture of external and internal (i.e., MCBIOS members), were responsible for evaluating each paper. Our goal for inclusion of papers in the conference proceedings remains the same as prior years: To be inclusive, yet rigorous in the peer-review process such that accepted papers are both high quality and reflective of the work presented at the conference. Papers generally fell into five categories:

## Genomic analysis

Michael Mayo *et al.* propose a one-dimensional diffusion-reaction model, in the form of a master equation, to analyze the non-equilibrium protein sliding kinetics along a segment of bacterial DNA [29]. Model validity is assessed through Monte Carlo simulations, and the results are interpreted within the context of bacterial transcription.

Sujoy Roy *et al.* describe a novel method to identify potential transcription factors from a list of differentially expressed genes using textual similarity between the genes and transcription factors reported in the literature [30]. Importantly, their approach has the potential to identify transcription factors that might be indirectly regulating the genes and, thus, would not be recognized by sequence-based analysis of the expressed gene promoters.

In “An Efficient and Extensible Approach for Compressing Phylogenetic Trees” [31], the authors demonstrate an efficient method for compressing large collections of Newick file representations of phylogenetic trees. To do this, the Newick file compression is directly operated on, without decompression needed.

## Network analysis

Ying Li *et al.* describe RefNetBuilder [32], a platform for the construction of putative reference pathways or gene regulatory networks from expressed sequence tags (ESTs), which provides a bioinformatics tool for researchers who work with non-model organisms.

Analyses of gene expression networks require understanding of how these networks can be manipulated to achieve desirable phenotype. The paper by Noushin Ghaffari *et al.*[33] proposes an alternative method for devising intervention/control policies for Boolean networks. Not only does it outperform other available techniques (MFPT-CP and SSD-CP) but eliminates the step of network reduction, thus simplifying analysis of large gene regulatory networks without loss of information.

Mohammad Shahrokh *et al*. [34] describe an algorithm for identifying a set of mutations that drove a healthy network into a cancerous state. Based on partial knowledge of the underlying gene regulatory network and the steady state distribution of the gene expression values in a given tumor, their algorithm is able to reconstruct the actual path of tumor progression in simulated and real networks.

## Systems biology

Fan Zhang and Jake Chen develop a repository for human organ-specific biomarkers called HOMER [35]. The ability to distinguish the tissue-specificity or enrichment of individual proteins is an important step in being able to define, molecularly, what is “normal” versus diseased and opens up avenues to use these patterns for diagnostics.

Xiaoning Qian and collaborators describe an improved method for computing the similarity between nodes when aligning different biological networks [36]. A semi-Markov random walk framework is used to calculate global correspondence scores between all pairs of nodes in the networks. The effectiveness of the approach in recovering known pathways is demonstrated with both synthetic and microbial networks.

Venkata Swamy and colleagues [37] describe a k-votes method for integrating protein-protein interaction (PPI) networks constructed from different databases. Edges are included in the integrated network if they are included in at least k of the source networks. The authors evaluate the “goodness” of the resulting network for different values of k by clustering the integrated network and evaluating the resulting clusters using measures of modularity, similarity-based modularity, clustering score, and enrichment in biological annotations. Results indicate that a k-value of two provides the best results.

Emerging biological threats or epidemics can rapidly spread through out the world due to readily accessible and rapid transportation systems. Social networking data, news, and databases where hospitals and clinical laboratories deposit screening data world wide can be harnessed to assess potential outbreaks of human pathogens. Researchers at Oak Ridge National Laboratories [38] outlined a strategy for Biological Signature Identification and Threat Evaluation System (BioSITES). The authors focused on the principles for constructing a semantic knowledgebase capable of integrating diverse data repositories and data streams to use in constructing such a threat identification system.

## Microarray studies/RNAseq

With thousands of microarray experiments publicly available, researchers are increasingly interested in conducting meta-analyses of them, yet finding a way to make each dataset directly comparable is challenging because of the technical and experimental variation. To solve this, Dozmorov and Wren report on a semi-automated method to normalize thousands of microarray experiments from NCBI’s GEO database, to create a standardized subset of many or even all experiments [39]. Once these datasets can be directly compared, the authors also show that a meta-analysis of gene-gene co-expression patterns across 1-color microarrays can be used to accurately predict gene ontology categories [40]. In addition to enabling topic-centric transcriptional meta-analyses, global meta-analysis of gene-gene co-expression patterns has also been shown to be useful in predicting function and phenotype for genes [41,42].

Minjun Chen *et al*. from the Food and Drug Administration present an assessment of models to find biomarkers from microarray experiments that are predictive of clinical endpoints [43]. They find that an ensemble method (i.e., using a combination of results from different methods) outperforms expert-nominated models.

As the cost of sequencing declines, RNAseq is poised to replace microarrays for relative gene expression analysis. Ying Wang *et al*. [44] perform a quality evaluation of two RNAseq datasets looking at chicken transcriptome gene representation. They focused on determining the optimal number of reads needed for detection of increasing percentages of annotated genes. Their study looked at sequencing read length coupled to the number of reads needed for optimal detection of both abundant and rare transcripts. Their work provides a useful, orderly approach to evaluating the level of RNAseq coverage needed for a given percent of gene detection in organisms other than human with well annotated genomes.

Markovets and Herman have developed a novel tool, Transcriptome Analysis with Circos, TrAC, for comparative analysis of RNAseq short-read data [45]. To validate TrAC, the authors selected the TCA and glycolysis pathways for comparison of normal brain and cancer RNAseq results. They calculated the expression level for a quantitative digital signal and visual output of the topology of transcript coverage. They showed unique expression patterns for pathway markers between normal and cancer samples as a way to characterize the drastic metabolic shifts in these pathways known as the Marberg effect. However, TrAC is not pathway specific and has broad application to gene expression-related biological questions.

### Imaging and structural biology

Determining the effects of hypertension and related diseases on vascular structure is complicated by the difficulty of measuring arterial morphology non-invasively. Diedrich *et al.*[46] computationally measured tortuosity, or twistedness, of arteries in Magnetic Resonance Angiography images. They were able to differentiate between hypertensive and non-hypertensive populations using arterial tortuosity measures thereby validating its use determining underlying arterial morphology.

In “An Improved Border Detection in Dermoscopy Images for Density Based Clustering“ [47], the authors take their previously developed dermoscopic region classification method, DBSCAN, and improve a preprocessing step to have direct access to image information, and make use of color information (if present), with significant improvement in both speed and accuracy over the original DBSCAN.

A paper by Xiaofei Nan *et al.*[48] demonstrates a feasibility of splitting a problem domain into several non-overlapping sub-problems to simplify learning tasks for biological predictions. The authors provide a metric to rank domain information attributes according to their potential to reduce the uncertainty of a classification task. In comparison with other methods this approach enhances prediction performance of the classifier.

## Miscellaneous

Halil Bisgin *et al.* report a method to identify topics within pharmaceutical labels to compare their similarity in an automated, objective manner [49]. This helps enable identification of drugs with similar effects, safety concerns and adverse events. They find their method is accurate at grouping drugs in each of these capacities.

Stephen Winters-Hilt and colleagues [50] provide an overview of potential applications of NTD technology in biosensing. Applications include single nucleotide polymorphism (SNP) detection, targeted DNA re-sequencing, protein isoforms assays, and biosensing via antibody or aptamer couple molecules. They also describe a kit platform, the Nanoscope Kit, to increase the accessibility of the technology.

In “A Modified Stokes-Einstein Equation for A-beta Aggregation” [51], the authors argue that the standard Stokes-Einstein equation is insufficient to understand A-beta 42 protein diffusion, given its aggregation behavior, and demonstrate a modified form of the Stokes-Einstein equation with improved modeling performance.

## Future meetings

The Inn at Ole Miss on the Campus of the University of Mississippi in Oxford, MS will be the site of MCBIOS 2012. The 2011-2012 MCBIOS President is Dr. Doris Kupfer of the Federal Aviation Administration. Dr. Edward Perkins of the US Army Engineer Research and Development Center is now the President-elect. MCBIOS is a regional affiliate of the International Society for Computational Biology (http://www.ISCB.org). For information regarding MCBIOS and our future meetings, see http://www.MCBIOS.org.

## Competing interests

The authors have no competing interests to declare.

## Author contributions

All authors served as editors for these proceedings, with JDW serving as Senior Editor. All authors helped write this editorial.
